# The EUROmediCAT Network and Databases: A Resource for Pharmacovigilance in Pregnancy

**DOI:** 10.1002/pds.70360

**Published:** 2026-04-29

**Authors:** Helen Dolk, Christine Damase‐Michel, Joan Morris, Amanda Neville, Ester Garne, Sue Jordan, Anke Rissmann, Alessio Coi, Deirdre Folan, Dieuwke Broekstra, Jennifer M. Broughan, Lea Bruneau, Clara Cavero‐Carbonell, Elly den Hond, Miriam Gatt, Mika Gissler, Babak Khoshnood, Anna Latos Bielenska, Hedvig Nordeng, Ljubica Odak, Mary O'Mahony, Isabelle Perthus, J. Luke Richardson, Florence Rouget, Joanna Sichitiu, David Tucker, Natalya Zymak‐Zakutnya, Maria Loane

**Affiliations:** ^1^ School of Medicine Ulster University Londonderry UK; ^2^ Pharmacologie, Faculté de Médecine, Université Toulouse III Centre Hospitalier Universitaire, INSERM, Center for Epidemiology and Research in POPulation Health (CERPOP) Toulouse France; ^3^ Population Health Research Institute, St George's University of London London UK; ^4^ IMER Registry, Centre for Epidemiology and Clinical Research University of Ferrara and Azienda ospedaliera e universitaria di Ferrara Ferrara Italy; ^5^ Department of Paediatrics and Adolescent Medicine, Lillebaelt Hospital University Hospital of Southern Denmark Odense Denmark; ^6^ Faculty of Medicine, Health and Life Science Swansea University Swansea UK; ^7^ Malformation Monitoring Centre Saxony‐Anhalt, Medical Faculty Otto‐von‐Guericke‐University Magdeburg Magdeburg Germany; ^8^ Unit of Epidemiology of Rare Diseases and Congenital Anomalies Institute of Clinical Physiology ‐ National Research Council Pisa Italy; ^9^ School of Medicine, Londonderry Ulster University Londonderry UK; ^10^ University of Groningen, University Medical Center Groningen, Department of Genetics Groningen the Netherlands; ^11^ National Congenital Anomaly and Rare Disease Registration Service (NCARDRS), National Disease Registration Service, Data & Analytics, Transformation Directorate, NHS England Leeds UK; ^12^ The Reunion Registry of Congenital Malformations REMACOR and National Institute of Health and Medical Research INSERM Center for Clinical Investigation (CIC)^14 10^ Clinical Epidemiology, Department of Public Health and Research Support University Hospital of La Réunion Saint‐Denis France; ^13^ Rare Diseases Research Unit, Foundation for the Promotion of the Research in Healthcare and Biomedicine Valencia Spain; ^14^ Provincial Institute of Hygiene (PIH) Antwerp Belgium; ^15^ Directorate for Health Information and Research, Ministry for Health Valletta Malta; ^16^ Department of Data and Analytics THL Finnish Institute for Health and Welfare Helsinki Finland; ^17^ Academic Primary Health Care Centre, Region Stockholm Stockholm Sweden; ^18^ Department of Molecular Medicine and Surgery Karolinska Institute Stockholm Sweden; ^19^ Centre de Recherche Epidémiologie et Statistique ‐ Inserm Université de Paris, UMR 1153 DHU Risques et Grossesse Maternité Port‐Royal Paris France; ^20^ Chair and Department of Medical Genetics Poznan University of Medical Sciences Poznan Poland; ^21^ Pharmacoepidemiology and Drug Safety Group, Department of Pharmacy, Faculty of Mathematics and Natural Sciences University of Oslo Oslo Norway; ^22^ Children's Hospital Zagreb, Centre of Excellence for Reproductive and Regenerative Medicine Medical School University of Zagreb Zagreb Croatia; ^23^ Department of Public Health, Health Service Executive – South, St Finbarr's Hospital Cork Ireland; ^24^ Auvergne Registry of Congenital Anomalies (CEMC‐Auvergne) Service de Génétique Clinique, Centre de Référence des Maladies Rares, Université Clermont Auvergne, CHU Clermont‐Ferrand, CNRS, SIGMA Clermont, Institut Pascal Clermont‐Ferrand France; ^25^ UK Teratology Information Service, Newcastle upon Tyne Hospitals NHS Foundation Trust and UK Health Security Agency Newcastle upon Tyne UK; ^26^ Brittany Registry of congenital anomalies, CHU Rennes Univ Rennes, Inserm, EHESP, Irset (Institut de recherche en santé, environnement et travail) ‐ UMR_S 1085 Rennes France; ^27^ Department of Woman‐Mother‐Child Lausanne University Hospital (CHUV) Lausanne Switzerland; ^28^ CARIS, Public Health Knowledge and Research, Public Health Wales Swansea UK; ^29^ OMNI‐Net Ukraine Birth Defects Program Rivne Ukraine; ^30^ Institute of Nursing and Health Research Ulster University Belfast UK

**Keywords:** congenital anomaly, data sources, medication safety, pharmacovigilance, pregnancy, registries

## Abstract

**Background:**

The evidence gap relating to the risk of congenital anomalies (CA) associated with first trimester medication exposure in pregnancy is well recognized.

**Aims:**

We describe the EUROmediCAT network and databases, and the methodological approach to pregnancy pharmacovigilance.

**Material and Methods:**

Multidisciplinary expertise includes CA diagnosis and epidemiology, pharmacoepidemiology, pharmacology and teratology. The EUROmediCAT central database comprises standardized data from 19 EUROCAT CA registries in 14 countries, including more than 40 000 CA cases 1995–2021 with first trimester medication exposure data recorded, and a population coverage of 14.6 million births, growing by more than 650 000 births per year. The distributed database enables federated data analysis across eight countries which can link data from CA registries to electronic healthcare data, with population coverage of up to 900 000 births per year for linkage to maternal prescriptions, of which 300 000 births per year for linkage also to data on all births.

**Results:**

The databases have enabled a variety of study designs: case‐malformed control studies, cohort studies, disease cohort studies, signal detection studies, prevalence and ecological studies, and medication utilization studies.

**Discussion:**

A key strength is that studies of CA risk can address accurately the specificity of risk by type of CA.

**Conclusion:**

EUROmediCAT presents a unique data and expert resource for tackling the enormous evidence gap regarding the safety of medication during pregnancy.

## Plain Language Summary

There is a well‐recognized need for more evidence about the safety of medication use in early pregnancy to guide optimal medication choice. This paper describes the EUROmediCAT network and its databases, and how these databases can be used to generate evidence about medication safety in pregnancy. A single central database includes data from congenital anomaly registries in 14 countries of Europe, with information on medication exposure of each case. This covers a population of 14.6 million births 1995–2021, and is growing by more than 650 000 births per year. Databases accessible in eight countries link congenital anomaly registries to prescription data (covering over 900 000 births per year), and to all births (covering over 300 000 births per year). These data can also be shared for large scale research. A key strength of the EUROmediCAT network and databases is that particular attention is paid to accurate information on the risk of specific types of congenital anomaly. EUROmediCAT presents a unique data and expert resource for tackling the enormous evidence gap regarding the safety of medication during pregnancy.

## Key Points

- EUROmediCAT presents a unique data and expert resource for tackling the enormous evidence gap regarding the safety of medication during pregnancy.
- Multidisciplinary expertise includes congenital anomaly diagnosis and epidemiology, pharmacoepidemiology, pharmacology, and teratology.
- The central database comprises standardized data from EUROCAT congenital anomaly registries which record first trimester medication exposure, covering 14.6 million births 1995–2021 in 14 countries.
- The distributed database enables federated data analysis across eight countries where congenital anomaly registries can be linked to electronic healthcare data (prescription and births data), with coverage of up to 8.4 million births 1995–2021.
- A key strength of EUROmediCAT data is that research can accurately address risks for specific congenital anomalies.

## 1 Purpose

Congenital Anomalies (CA) are structural or functional anomalies that arise during intrauterine life, and may be identified before or at birth, or later in life. Teratogenic medications such as thalidomide, isotretinoin, and valproic acid [1] show us the importance of pregnancy pharmacovigilance in relation to the risk of CA, and the specificity of action of medication exposures in relation to specific CA [2]. The enormous evidence gap relating to the risk of CA associated with first trimester medication use is well established [3]. This leads to a precautionary approach limiting the access of pregnant women to medications which are essential for the effective treatment of their conditions, and makes it difficult for pregnant women and their clinicians to weigh the harms and benefits of different medicines when choosing the optimal treatment.

CA are rare events, especially specific types, which may occur in only one in 1000 births or less. Limited numbers often force researchers to group CA of heterogeneous etiology together, which may mask risks for specific CA, as teratogens rarely affect all organ system [2]. Pregnancy pharmacovigilance requires data relating to a large population, combining well documented CA diagnoses, with systematic recording of medication exposure during the sensitive period of embryonic development (within first trimester of pregnancy).

To meet this need, the EUROmediCAT network of researchers and databases is dedicated to research and surveillance regarding CA risk associated with maternal medication exposure during the first trimester of pregnancy. The network is an extension of the EUROCAT network for the registration and population‐based surveillance of CA [4]. The EUROmediCAT network is a multidisciplinary collaboration of EUROCAT registries (which draw in clinical expertise in CA diagnosis, classification, and ascertainment, and expertise in CA epidemiology and available local healthcare databases) and partners who have medication‐related expertise (pharmacology, pharmacoepidemiology and human teratology).

The aim of this paper is to describe the EUROmediCAT network and databases, and the methodological approach to the use of these databases for pregnancy pharmacovigilance purposes. We aim thereby to encourage the future use of this data and expert resource.

## 2 Data Description

### 2.1 Overview of Databases and Population

**FIGURE 1:**
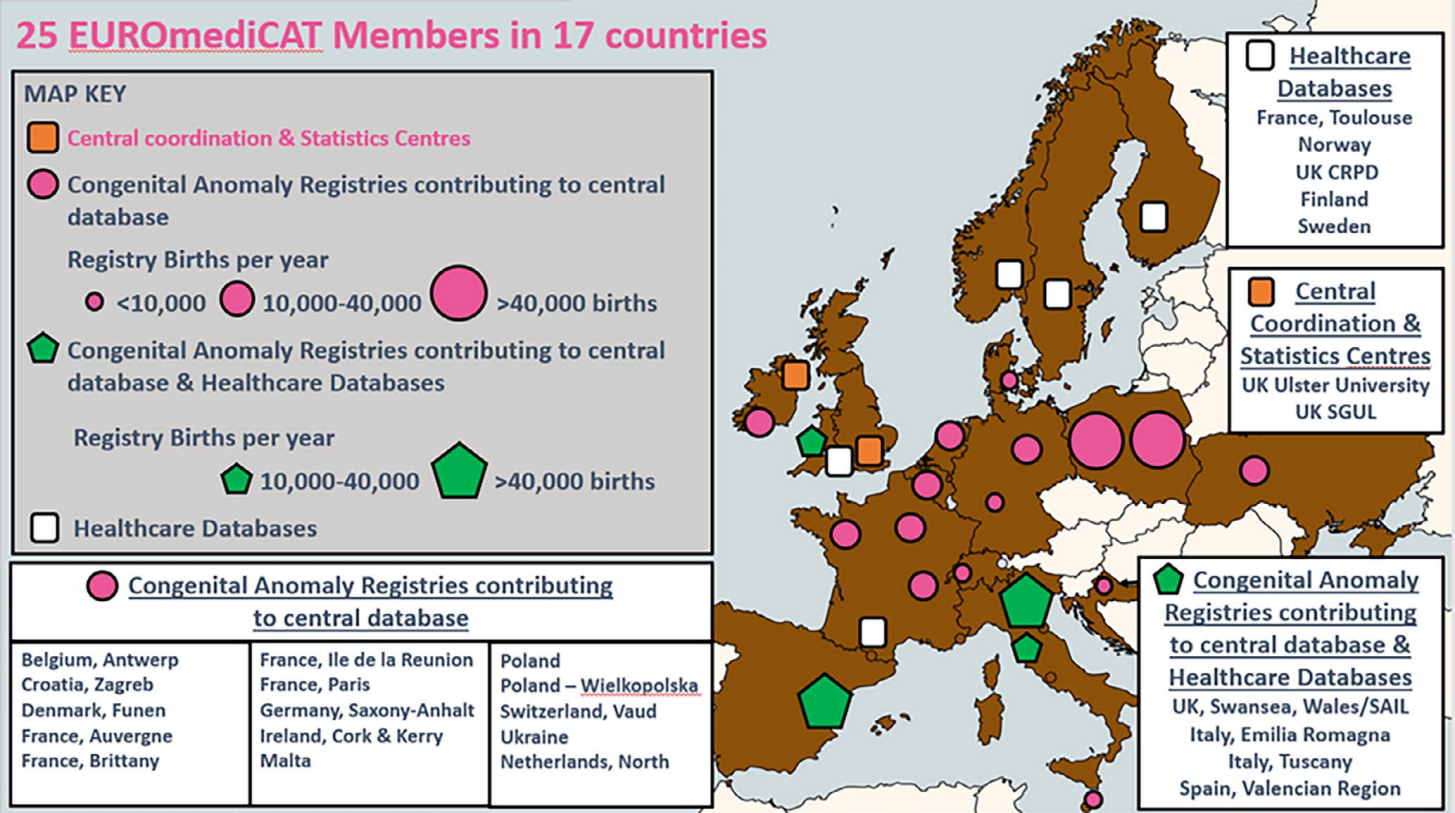
Map of EUROmediCAT Registries.

EUROmediCAT has a *central database* of individual anonymized cases of CA contributed by 19 EUROCAT registries in 14 countries (Table 1, Figure 1) that is, all EUROCAT registries which record both CA diagnosis and medication exposure. Registries upload CA data annually via a secure portal, with a 19 month delay from the birth year. For example, CA cases born in birth year 2021, and diagnosed up to at least age one by the end of 2022, were transmitted to the central database in October 2023. The EUROmediCAT population‐based central database covered 14.5 million births from 1995 to 2021 (transmitted by 2023), and is growing by approximately 650 000 births per year (Table 1).

**TABLE 1:** Registries and countries participating in central and distributed database, with years, population size and total CA cases with medication exposure (excluding vitamins, minerals, and folic acid) recorded.

Registry (country)	Data available from	Approx annual no. births^a^	Total no. births (all available years 1995–2021)	Total CA cases with first trimester medication exposure^a^, ^b^ (all available years 1995–2021)	Central (1) or distributed^c^ (2, 3) databases
Funen (Denmark)	1995	4900	138632	669	1, 2, 3
Paris (France)	2001	23300	518539	2025	1
Isle de la Reunion (France)	2005	13150	228378	698	1
Brittany (France)	2011	32450	375738	2404	1
Auvergne (France)	2011	12250	142052	1244	1
Tuscany (Italy)^d^	1995	21850	730394	1972	1, 2
Emilia Romagna (Italy)	1995	30350	899966	3465	1, 2, 3
N Netherlands (Netherlands)	1995	16100	479573	5792	1
Vaud (Switzerland)	1997	8950	197944	954	1
Zagreb (Croatia)	1995	6950	142525	348	1
Malta (Malta)	1996	4500	111506	979	1
Antwerp (Belgium)	1997	19150	483882	1354	1
Saxony‐Anhalt (Germany)	2000	16100	380941	2059	1
Cork and Kerry (Ireland)	1996	8350	229690	679	1
Wales (United Kingdom)^d^	1998	28100	786435	4882	1, 2, 3
Ukraine (Ukraine)	2009	20050	349761	833	1
Wielkopolska (Poland)	1999	33650	851951	929	1
Poland (Poland)	1999	320550	6840733	4988	1
Valencian Region (Spain)	2007	36150	650355	4045	1, 2, 3
Registries not in central database					
Finland (Finland)	1996	49725	1443400	21395	2, 3
Haute‐Garonne (France)^e^, ^f^	Mid 2004	10000	169149	2909	2, 3
Norway (Norway)^e^	2004	56100	869483	16032	2, 3
Sweden (Sweden)^e^	1999	113250	2458700	NA	2, 3
England (UK)^e^, ^g^	2021	580000	580000	2362	2
All registries		1452700	20059727	83017	1 or 2 or 3
All registries in Central Database		656850	14538995	40 319	1
All registries in Distributed Database with linkage to prescription records		917200	8726514	57731	2
All registries in Distributed Database with linkage to prescription records and all births		315350	7416120	53397	3

^a^ Based on most recent available year in 2024 which was 2021 for all registries except Paris (2020), Iles de la Reunion (2020), Poland (2020), Haute Garonne (2020), Norway (2018), Zagreb (2017).

^b^ Excludes vitamins, minerals, and folic acid (FA)—ATC (Anatomical Therapeutic Chemical Code) codes A11, A12, B03B, and B03A.

^c^ 1 = central 2 = CA registry linked to prescription data, CA cases only 3 = CA registry linked to all births and to prescriptions for all births. Note that distributed (linked) data nearly always requires extra permissions and payment to conduct linkage.

^d^ Tuscany linked prescription data is only available for the years 1995–2012; Wales is able to link EUROCAT CA data to maternal prescriptions for ~85% of people.

^e^ These registries contribute to selected specific studies.

^f^ Haute‐Garonne (EFEMERIS) is the only center which does not have a EUROCAT registry, but collects equivalent data, which is translated to the EUROCAT Common Data Model.

^g^ England has a EUROCAT CA registry linkable to prescription data. Medication utilization studies also access the CPRD primary care data [5, 6, 7].

EUROmediCAT also has a *distributed database* in 10 centers in eight countries (Table 1, Figure 1). Some of these members also contribute to the central database. The general principle of a distributed database is that individual patient data do not leave the country of origin, and instead federated analysis [8] takes place. The distributed database includes CA registries which can be linked to prescription databases (10 centers in eight countries, covering over 900 000 births per year), and CA registries which can link to all births in the source population as well as to prescription data (eight centers, covering over 300 000 births per year) (Table 1). Those registries which have data on all births may have Medical Birth Registries (in Nordic countries) or link maternity/obstetric or birth registration data to CA registry data (Wales, Emilia Romagna, and Valencian Region) or maintain a linked pregnancy (mother‐baby) cohort (EFEMERIS in Haute‐Garonne in France, and Finland).

### 2.2 Congenital Anomaly Registries

The characteristics of EUROCAT registry data are shown in Table 2. EUROCAT CA registries include all cases of CA diagnosed among livebirths and fetal deaths from 20 weeks gestational age, as well as terminations of pregnancy for fetal anomaly (TOPFA) at all gestations. The complete ascertainment of TOPFA by registries is essential, as they can constitute a large proportion of certain anomalies—11% for all major anomalies combined since 1995, and over 40% for neural tube defects and omphalocele (Table 3).

**TABLE 2:** Characteristics of EUROCAT registry data (6).

Characteristic	Comment
Population‐based—register all CA cases occurring among births to mothers resident in a defined geographic area.	The population‐based approach helps prevent selection bias related to hospital referral (in utero after prenatal diagnosis, or postnatal) for specialist care.
Register all cases of CA diagnosed among livebirths and fetal deaths from 20 weeks gestational age, as well as terminations of pregnancy for fetal anomaly (TOPFA)	Spontaneous abortions less than 20 weeks are excluded as they are not well recorded nor routinely examined for CA. Inclusion of TOPFA important for severe anomalies where TOPFA are frequent, to avoid bias and increase statistical power.
Include diagnoses up to at least 1 year of age or beyond^a^	Important for accurate diagnostic data (e.g., diagnosis of genetic syndromes), and for ascertaining congenital heart defects and other CA that are not externally visible at birth and may be detected later.
Common Data Model—a set of standard variables with standardized coding [9]^b^	Structural anomalies and syndromes coded with International Classification of Diseases (ICD v10) codes, with one digit Royal College of Paediatrics and Child Health (RCPCH) extension; data about the baby (e.g., type of birth, birthweight, gestational age); data about the mother (e.g., age, illnesses before and during pregnancy, medications taken in first trimester).
Maternal medication exposure in the first trimester (from the 1st day of the last menstrual period up to the 12th week of gestation [9]) is coded using full ATC codes [10].	Free text allows specification of dose and exact timing, where known.
Standard EUROCAT CA subgroups [9, 11].	A standard computer program assigns cases to these binary subgroups according to ICDv10‐RCPCH codes.
EUROCAT CA subgroups exclude minor anomalies which have in themselves little medical or functional impact. There is a standard EUROCAT list of minor anomalies excluded [9].	Minor anomalies tend to be common and very variably recorded, and their inclusion leads to statistical noise. Includes patent ductus arteriosus (PDA) in preterm babies, which is relatively common and indicates an immature heart. Other heart anomalies which can spontaneously resolve, such as small atrial septal defects (ASDs), are included if verified as persisting at 6 months.
A multiple congenital anomaly (MCA) classification [9, 11], initially by computer algorithm, with confirmation of uncertain cases by manual review, assigns cases to whether they have an isolated anomaly or MCA, or a syndrome with a known genetic or environmental cause.	Isolated anomalies include “sequences” where the initial anomaly leads to others, e.g., spina bifida which leads to clubfoot, assigned to the primary anomaly.

^a^ Paris EUROCAT registry registers cases diagnosed up to 1 week of age.

^b^ The Common Data Model is given in Appendix 1, and links to the proportion of missing data per variable given in Appendix 2.

**TABLE 3:** Prevalence of selected^a^ CA subgroups per 10,000 births, all EUROmediCAT Registries Contributing to Central Database Combined, 1995–2021 (total population coverage 14.5 million births).

CA subgroup^a^	Congenital anomaly prevalence LB + FD + TOPFAper^b^, ^c^ 10,000 births	% TOPFA^c^ per CA subgroup	%Genetic^d^ per CA subgroup
All anomalies	225.3	11%	16%
Nervous system anomalies	20.1	32%	14%
Neural tube defects	7.3	48%	5%
Anencephaly	2.1	71%	3%
Spina Bifida	4.3	37%	5%
Hydrocephaly	4.2	29%	13%
Severe microcephaly	2.4	8%	19%
Eye anomalies	3.9	5%	21%
Ear, face and neck anomalies	2.6	8%	19%
Congenital heart defects	78.1	5%	13%
Severe congenital heart defects	19.4	12%	21%
Cleft lip with or without cleft palate	8.1	6%	8%
Cleft palate	5.8	3%	12%
Esophageal atresia with or without trachea‐oesophageal fistula	2.1	5%	10%
Ano‐rectal atresia or and stenosis	2.7	11%	10%
Diaphragmatic hernia	2.1	14%	10%
Gastroschisis	1.6	13%	2%
Omphalocele	2.3	43%	32%
Urinary system	30.4	8%	6%
Hypospadias	16.3	0%	2%
Limb reduction defects	5.1	16%	12%
Club foot–talipes equinovarus	9.6	8%	7%
Craniosynostosis	2.3	4%	12%
Vascular disruption anomalies	5.8	14%	3%

^a^ Coding and order of subgroups according to EUROCAT Guide 1.4. JRC‐EUROCAT‐Full Guide 1 4 version 22‐Nov‐2021.pdf (europa.eu). For a full list of CA subgroups, see EUROCAT Guide 1.4. Note that EUROmediCAT will soon be moving to EUROCAT Guide 1.5. For the prevalence of all EUROCAT CA subgroups see Prevalence charts and tables|EU RD Platform (europa.eu).

^b^ Cases include livebirths (LB), fetal deaths from 20 weeks gestation, and TOPFA.

^c^ Prevalence and % TOPFA include cases with multiple anomalies.

^d^ Cases where the anomaly is part of a diagnosed genetic syndrome. Genetic cases are usually excluded in EUROmediCAT studies.

The coding of CA, and delineation of different types of CA, is complex, so the EUROCAT subgroup allocation [9, 11] brings great added value and harmonization. The prevalence of selected EUROCAT subgroups is shown in Table 3. Genetic syndrome cases, a significant proportion of some types of CA (Table 3), are excluded from studies of medication exposure in pregnancy, or used as controls. Genetic syndromes make up 16% of all cases, and up to one third of specific CAs (Table 3). By concentrating on well‐defined and specific CA subgroups, excluding a standard list of minor anomalies (Table 2), studies can achieve greater statistical power to detect genuine risks.

### 2.3 Medication Exposure Data Sources

The central database contained 40 319 CA cases to birth year 2021 with one or more first trimester medication exposures recorded, excluding vitamins and minerals (Table 1). The number and proportion of cases in the central database by type of medication (to 2nd level ATC [10]) are shown in Table 4. The frequency of recording is determined by the frequency with which the medication is taken and the likelihood that it would be recorded in the medical records. Overall, thyroid medications are the most frequently recorded medication, at 2.2% of CA cases and 17.9% of all first trimester exposed CA cases (Table 4) due to a combination of their frequency of use and their relevance to antenatal care, ensuring high levels of recording.

**TABLE 4:** Number and % of cases exposed to each ATC medication category in first trimester, and prevalence per 1000 CA cases, all EUROmediCAT Registries Contributing to Central Database Combined, 1995–2021 combined.^a^

ATC code (2nd level)	ATC therapeutic subgroup	No. CA cases with first trimester exposure recorded	% of all exposed	Prevalence per 1000 CA cases
HO3	Thyroid therapy	7260	17.93%	22.22
GO3	Sex hormones	6229	15.38%	19.06
NO2	Analgesics	5902	14.58%	18.06
JO1	Antibacterials for systemic use	5384	13.30%	16.48
RO3	For obstructive airway diseases	2946	7.28%	9.02
A10	Used in diabetes	2500	6.17%	7.65
BO1	Antithrombotic agents	2170	5.36%	6.64
NO6	Psychoanaleptics	2031	5.02%	6.22
AO2	For acid related disorders	2027	5.01%	6.20
RO6	Antihistamines for systemic use	1940	4.79%	5.94
AO3	For functional gastrointestinal disorders	1682	4.15%	5.15
NO5	Psychoeleptics	1344	3.32%	4.11
GO2	Other gynecologicals	1338	3.30%	4.10
HO2	Corticosteroids for systemic use	1208	2.98%	3.70
NO3	Aniepileptics	1178	2.91%	3.61
GO1	Gynecological antiinfectives & antiseptics	1174	2.90%	3.59
CO7	Beta blocking agents	889	2.20%	2.72
CO2	Antihypertensives	876	2.16%	2.68
MO1	Antiinflammatory & antirheumatic products	845	2.09%	2.59
RO1	Nasal preparations	725	1.79%	2.22
CO8	Calcium channel blockers	519	1.28%	1.59
AO6	For constipation	515	1.27%	1.58
DO7	Corticosteroids, dermatological preparations	456	1.13%	1.40
NO1	Anesthetics	409	1.01%	1.25
RO5	Cough & cold preparations	407	1.01%	1.25
CO5	Vasoprotectives	381	0.94%	1.17
C10	Lipid modifying agents	378	0.93%	1.16
AO7	Antidiarrheals, intestinal agents	366	0.90%	1.12
DO1	Antifungals for dermatological use	342	0.84%	1.05

^a^ Total population 14.5 million births; total CA cases = 326759; total CA cases with recorded first trimester medication exposure 40319.

Registries contributing to the central database use one or more of four sources of medication exposure information (Table 5), the first three sources including both prescribed and over‐the‐counter medications:

– *Prenatal maternity/obstetric records*—medications recorded by midwives and obstetricians in the course of care given to pregnant women, which includes specialist care for high‐risk women. The information may come from maternal report or recording of healthcare given. For example, in Funen (Denmark), mothers fill out a questionnaire at 6–9 weeks gestation, before their first antenatal visit, including medication intake, and the antenatal record includes the referral letter from the General Practitioner to the hospital, which documents all prescribed medications.
– *Postnatal medical records* of the baby which record what medication the mother reports she had taken during pregnancy, for diagnostic and healthcare purposes e.g., records of clinical geneticists.
– *Postnatal interviews of mothers* conducted for CA surveillance purposes. Seven registries conduct postnatal interviews of mothers of CA cases (Table 5). Some registries also interview mothers of non‐CA control babies, but this information is not transmitted to the central database.
– *Prescription records*, obtained on a case‐by‐case basis. For example, in Northern Netherlands, mothers are asked permission for their prescription records to be accessed and are, in addition, interviewed as to whether they took the medication prescribed [12].

**TABLE 5:** Data sources for medication exposure, by registry, with % CA cases with medication recorded in first trimester.

Registry	% CA cases with medication recorded in first trimester, excluding vitamins, iron and folic acid	Medication exposure data sources contributing to central database				Linked prescription data available for specific studies
		Prenatal records^a^	Postnatal records^b^	Postnatal interview	Prescription records	
Registries contributing to central database						
Funen (Denmark)	17%	Y	Y	N	N	Y
Paris (France)	12%	Y	Y	Y	N	Y
Tuscany (Italy)	13%	Y	Y	Y	N	Y^c^
N Netherlands (NL)	42%	Y	Y	Y	Y	N
Emilia Romagna (Italy)	17%	Y	Y	Y	Y	Y^c^, ^d^
Vaud (Switzerland)	13%	Y	Y	N	N	N
Zagreb (Croatia)	13%	Y	Y	N	N	N
Malta (Malta)	28%	Y	Y	N	N	N
Antwerp (Belgium)	10%	Y	Y	N	N	N
Saxony‐Anhalt (Germany)	17%	Y	Y	Y	N	N
Cork and Kerry (Ireland)	11%	Y	Y	N	N	N
Wales (UK)	17%	Y	Y	N	Y	Y^e^
Auvergne (France)	25%	Y	Y	N	N	N
Ukraine (Ukraine)	9%	Y	Y	N	N	N
Isle de la Reunion (France)	10%	Y	Y	N	N	N
Wielkopolska (Poland)	4%	Y	Y	Y	N	N
Poland (Poland)	5%	Y	Y	Y	N	N
Valencia Region (Spain)	24%	Y	Y	N	Y	Y^d^, ^f^
Brittany (France)	17%	Y	Y	N	N	N
Registries contributing to distributed database only						
Finland (Finland)	31%	—				Y
Haute‐Garonne (France)	75%					Y^g^
Norway (Norway)	37%					Y
Sweden (Sweden)	NA					Y^d^
England (UK)^h^	17%					Y

^a^ Includes hospital antenatal records (all), referral letters from GP to maternity hospital (Denmark), primary care records (Valencia).

^b^ Includes any child health records, clinical genetics, pediatric records etc.

^c^ Tuscany linked prescription data is only available for the years 1995–2012; For Emilia Romagna 2008–2020, 53% of cases were exposed to any medication in the first trimester by linkage to prescription data.

^d^ Prescription data not available for TOPFA, therefore all TOPFA (exposed or unexposed) are excluded from analysis.

^e^ Wales: Full linkage to primary care prescribing data for 86% of the population is available via SAIL. For 1998–2020, 45% of cases were exposed to any medication in the first trimester by linkage.

^f^ Valencia: The Integral Management of Pharmaceutical Services (GAIA) which registers the prescription and dispensing of drugs in ambulatory (outpatient) care. From 2013 to 2019, 47% of cases were exposed to any medication in the first trimester by linkage.

^g^ Haute‐Garonne: medication exposure data comes from linkage to prescription data as well as linked maternity records.

^h^ England: 87% of CA cases with Estimated Date of Delivery recorded to allow timing of prescription to be calculated. Proportion of unlinked cases not known.

Table 5 shows the proportion of CA cases exposed to first trimester medication, excluding vitamins and minerals, per registry. The highest recorded proportion in the central database is 42% in Northern Netherlands, which has the most comprehensive exposure ascertainment. The lowest proportion is in Poland, with only 4%–5% of CA cases with recorded medication exposure.

Registries contributing to the distributed database link CA cases to prescription data (either issued, from primary care data, or dispensed, and from pharmacy data). CA registries include date of birth and gestational age, which in combination with the date of prescription allows the gestational age at prescription to be calculated. This systematically includes even prescriptions in the very early stages of pregnancy, relevant to organogenesis, before the pregnancy has been recognized. It does not, however, indicate the date on which the medicine was taken, or if it was taken at all. Patterns of repeat prescribing offer insight into medication adherence. Data on dose varies between countries, as quantity dispensed, rather than daily dose, is more relevant to reimbursement. In some regions/countries, linkage with prescription data is not possible for TOPFA (Table 5), requiring therefore the exclusion of all TOPFA from analysis. Prescription data, furthermore, do not include over‐the‐counter medication, internet medication purchases, or medications prescribed/administered in hospital rather than in the community, and may not include private prescriptions.

Among registries contributing to the distributed database that collect data by linkage to prescription records, first trimester medication exposure is recorded in 17% of CA cases in England, 31% of CA cases in Finland, 37% in Norway, and 75% in Haute‐Garonne (Table 5), reflecting national differences in medication use, in what is obtained by prescription rather than over‐the‐counter, in which medications are covered by insurance, and in completeness of data linkage.

A validation study comparing medication recorded by registries to linked prescription records, found that medications for chronic diseases, which place women in a high‐risk category in pregnancy, are well recorded in registry data sources [13], and this was supported in a validation of recording of antiseizure medication reporting [14]. However, medications for acute or episodic diseases, such as antibiotics, are recorded much less completely [13]. The completeness of exposure ascertainment from registry data sources varies considerably by registry [13].

It is vitally important that maternity units should review and, where necessary, improve their recording of medications taken, to improve pregnancy pharmacovigilance.

### 2.4 Data Sharing Procedures

EUROmediCAT has used two main approaches to multicentre data sharing: a central database and a distributed database.

**FIGURE 2:**
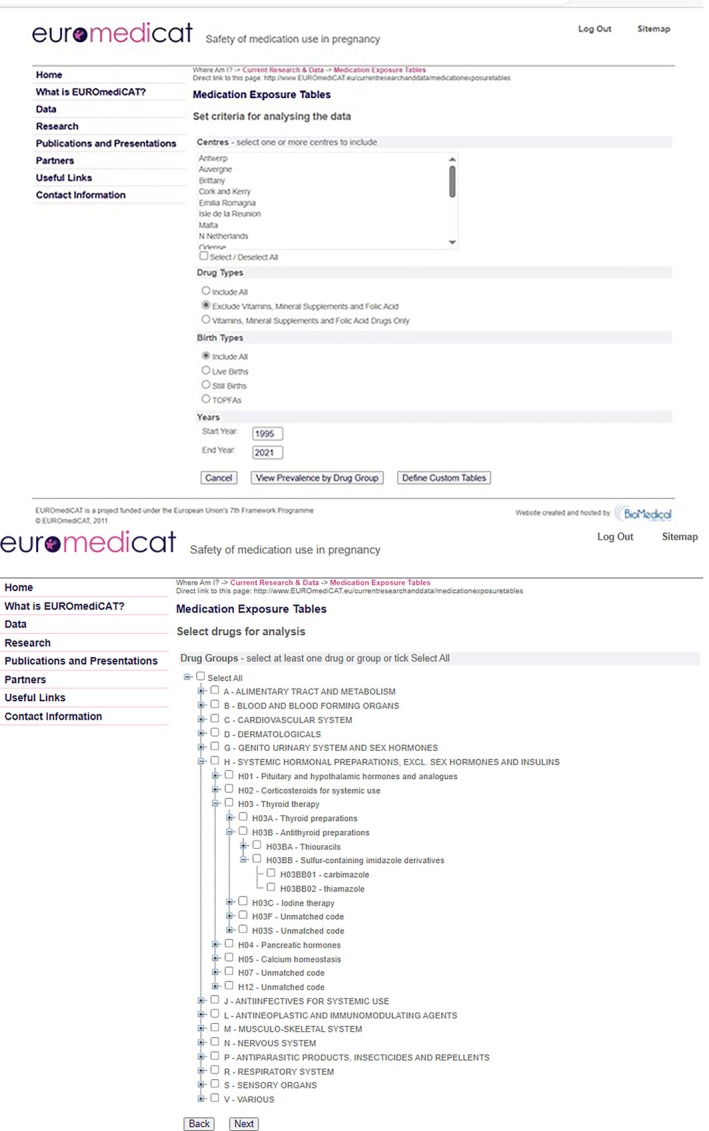
Screenshot of medication prevalence tables on website (members only).

The central database uses the EUROCAT Common Data Model (CDM) which covers both the congenital anomaly diagnostic variables, medication exposure, and other variables (Appendix 1). The strengths of this data sharing approach (Table 6), benefiting from the existence of the parent EUROCAT network, are that (i) individual patient data [IPD] are available for complete statistical exploration, (ii) the data are updated annually and ready for any new study which requires only a single ethics approval, (iii) a protocol can quickly be prepared including the recorded prevalence of the medication exposure in the database (Figure 2), (iv) only one statistical programmer is required for a study, (v) the marginal cost of each study is lower, and (vi) all data partners regularly collaborate with extensive mutual understanding of data and national differences. However, where the data source involves data linkage, linked data cannot be shared across national boundaries, and requires a federated approach with a distributed database, with ethics approval in each participating centre for each study.

**TABLE 6:** *C*omparison of characteristics of EUROmediCAT central database and distributed database.

Characteristic	Central database	Distributed database
Max population coverage per year (births)	650 000 in 14 countries	300 000 where CA register is linked to all births/pregnancies + prescriptions; 900 000 linked to prescriptions only; in eight countries.
Data linkage required	No	Yes
Individual Patient Data (IPD) available centrally	Yes. Full statistical data exploration possible.	No. Sharing of aggregate numbers and meta‐analysis of pre‐specified parameter estimates
Size and nature of variable set	Subset of EUROCAT variables (Appendix 1)	All healthcare generated variables potentially available; chosen variable set may vary across studies
Common data model available	Yes	Partial (CA register only)
Use of Malformed controls in case–control studies	Yes	Yes
Use of non‐malformed births in case–control or cohort studies	No	Yes, centres where CA register is linked to all births/pregnancies
Ease of use for signal detection studies (across all medications)	Straightforward and rapid due to IPD availability	Complex and requires significant system preparation, but not insurmountable.
Ease of use for hypothesis testing	Straightforward and rapid due to IPD availability	Complexity and timescale depend on number and readiness of participating centres
Includes prescription data	No (some exceptions–Table 5)	Yes
Includes OTC data	Yes	Some centers—if linking pregnancy data from maternity care
Data lag (from birth year (n) to data available in database)	October of year (*n* + 2) to allow all babies to reach at least 1 year of age for diagnosis + data preparation; central ethics approval.	Minimum: calendar year (*n* + 2) to allow all babies to reach at least 1 year of age for diagnosis + data preparation; ethics approval in each center required.
Data Sharing Requests	Request to EUROmediCAT Steering Group with protocol, followed by individual registry permission (typically 4 month process).	Request to EUROmediCAT Steering Group with outline protocol, followed by distributed database partners and detailed protocol development, followed by request to authorised data holders in each country (time depends on complexity).
Statistical programmer required	Centrally	Each participating centre/database, though program templates may be written centrally.
Data cost	30000 euros including local registry data verification where necessary.	Allow average 30 000 euros per cente participating for linked data, depending on study specification

Federated analysis of a distributed database relies on all participating databases converting their data into a CDM. For the first EUROmediCAT studies analyzing the distributed database [15, 16], in addition to using the EUROCAT CDM, a study‐specific CDM extension was created in the protocol, and each of the three participating data providers converted their data to this CDM and provided counts and parameters for meta‐analysis. Output files (tables) are subject to disclosure control for suppression of small possibly disclosive numbers, but permission is usually obtained for data sharing of small numbers if they are to be transmitted securely and combined before publication. This simple federated approach is responsive to changes over time in databases, and fully utilizes the expertise of local data experts. However, it requires statistical programmer time and availability in participating data centres.

Subsequently, many EUROmediCAT partners have contributed to the IMI ConcePTION project. The CDM adopted [17] stopped at syntactic harmonization and has proved a slow process to implement. It was not tested in full medication safety studies relating to congenital anomaly risk.

The plan in EUROmediCAT is to develop an extended CDM for the distributed database limited to the variables needed for standard medication safety studies relating to congenital anomalies (see Section 3), building on the EUROCAT CDM and the extended CDM of the sister project EUROlinkCAT [18], which should be quick to implement and relatively easy for new data partners to join. Partners would convert their data to this CDM prior to running centrally created programs for each study.

EUROmediCAT has also applied to join DARWIN EU [19], which uses the Observational Medical Outcomes Partnership (OMOP) CDM, but the pregnancy‐specific module for OMOP is still under development [20].

We believe that the active collaboration of local experts with experience in their pregnancy databases and the local healthcare context is essential for successful federated data analysis, appropriate data interpretation and flexibility to changing databases. The EUROmediCAT network provides ongoing collaboration and trust, and the contribution of varied disciplinary expertise, with regular meetings and collaborative studies.

Table 6 compares the characteristics, strengths and limitations of a central database approach versus a federated analysis of distributed database approach. The advantages of a distributed database approach are that (i) it can include partners which are not able to transmit data to the central database (mainly due to data protection restrictions related to data linkage), (ii) it has a wider range of data variables potentially available as a result of data linkage, (iii) it has the potential to include all births in the source population and thus conduct whole‐population cohort studies, and (iv) linkage with prescription data results in considerably higher prescribed medication exposure ascertainment. The limitations relate to cost and complexity.

### 2.5 Governance and Access to the EUROmediCAT Databases

Governance procedures are agreed by the EUROmediCAT Steering Group (EMSG). All registries contributing to the central database have local ethical approval to upload anonymized, standardized CA data to a secure portal which are downloaded and imported into the central database by staff at the EUROmediCAT Co‐ordinating Centre, Ulster University (UU). Data are transmitted and processed according to the General Data Protection Regulations (GDPR). UU ethics committee has given approval to hold the central database, periodically reviewed. A Memorandum of Agreement details governance procedures for all registries contributing data to the central database. The data continue to be owned by the contributing registries and are used only with their permission. All registries are invited to collaborate in studies using the central database, contributing both data and expertise.

Researchers can apply to the EMSG for data by submitting an application form and protocol https://www.euromedicat.eu/research/howtoproposeorcommissionspecificstudies. These are reviewed and approved by the EMSG (meeting every 3 months), following which individual registry consent is obtained (typically taking 1 month). External researchers can either apply to collaborate with the network or commission a study. International Committee of Medical Journal Editors (ICMJE) authorship rules apply, and all participating EUROmediCAT registries are co‐authors responsible for the correct interpretation of their data. The cost of a study depends on the data requirements. Use of the distributed database is considerably more expensive than use of the central database, as it requires data linkage in each participating country (Table 6).

EUROmediCAT was established as a network in 2011 with European Union Framework 7 funding. Since then, there have been diverse funding sources—national research funding, European Medicines Agency (via EUROmediSAFE), EU IMI2 funding (via ConcePTION consortium), doctoral studentships, and pharmaceutical companies. Where funding comes from the pharmaceutical industry, the EncePP Code of Conduct for Scientific Independence and Transparency is followed [21]. This ensures that pharmaceutical companies are not involved beyond protocol finalization, and the research team is free (and obligated) to publish the results. The lack of a source of core long term funding for EUROmediCAT is an ongoing challenge.

## 3 Study Designs

EUROCAT has always engaged in monitoring the prevalence of specific CA over time, via statistical monitoring of trends and clusters [22, 23]. This approach is limited to detecting risks associated with widely used medications with high relative risks. EUROmediCAT expanded this methodology to analyse individual‐level data on medication exposure.

EUROmediCAT studies distinguish signal detection (hypothesis generation) from signal (hypothesis) testing. A comprehensive literature review first documents the previous signals for any specific medication of interest. These are subject to a signal testing analysis. Signal detection analyses adjust for multiple testing due to the many different types of CA and medication being analysed.

EUROmediCAT studies usually either (a) Investigate a medication class, rather than a single medication or product, including comparisons between the medications within the class e.g., SSRIs, antiseizure medications, antiasthmatics [14, 24, 25, 26, 27, 28] or (b) Investigate a particular congenital anomaly in relation to all recorded medication exposures for example, Ebstein's anomaly, gastroschisis and eye anomalies [29, 30, 31].

This provides the context that focusing uniquely on the initial signal cannot provide that is the suspected association specific to one or more anomalies or to one or all medications within the class? The evidence thus provided is more useful for assessing treatment options and differs from the single medicine approach of post authorization safety studies by market authorization holders as part of their regulatory requirements.

### 3.1 Case‐Malformed Control Studies

A number of “case‐malformed control” (CMC) studies have been conducted using the central database, relating to antiepileptic, antiasthmatic, antibiotic medications, beta‐blockers, methadone and metformin [14, 24, 25, 26, 27, 28, 29, 30, 31, 32, 33, 34, 35]. Exposure data can also be enhanced by linkage to prescription data [13] (Table 1). CMC studies are effective for signal testing, where the specific CA(s) identified in a previous signal(s) are compared to all other CA (malformed controls) in terms of the proportion exposed to the medication of investigation that is they measure specificity of the association.

Malformed controls exclude CA at a hierarchical level above the case subgroup (e.g., if spina bifida is the case subgroup, it is compared to all other CA excluding neural tube defects). Genetic syndromes are either excluded (from both cases and controls) or used as a second control group. Among non‐genetic malformed controls, an exploratory hypothesis‐generating (signal detection) investigation is also performed, comparing the proportion exposed to the medication in each EUROCAT CA subgroup to all other malformed controls, adjusting for multiple testing. Given the variable nature of medication exposure prevalence and reporting, and the variation in CA prevalence and reporting, data analyses always stratify by registry or conduct equivalent meta‐analysis approaches.

The CMC study is a classic epidemiological case–control design [32, 36, 37, 38], originally recommended to overcome recall bias, although most registry exposure data is prospectively recorded and thus not subject to recall bias. CMC studies are efficient as they do not require data collection on babies without CA. An unbiased estimate of the Odds Ratio should be provided, since the information on exposure is collected in exactly the same way for both cases and controls. By including a wide range of malformations in the control group, “teratogen non‐specificity bias”, where the control group may itself be associated with the medication under study, leading to an underestimate of risk, may be avoided [32, 36, 37, 38]. This bias can also be reduced by excluding from malformed controls any CA subgroup identified in previous signals. The use of a second genetic control group is an additional strategy to assess this potential bias.

The protocol further includes a data verification stage, where registries verify individual data on exposed cases and controls.

**FIGURE 3:**
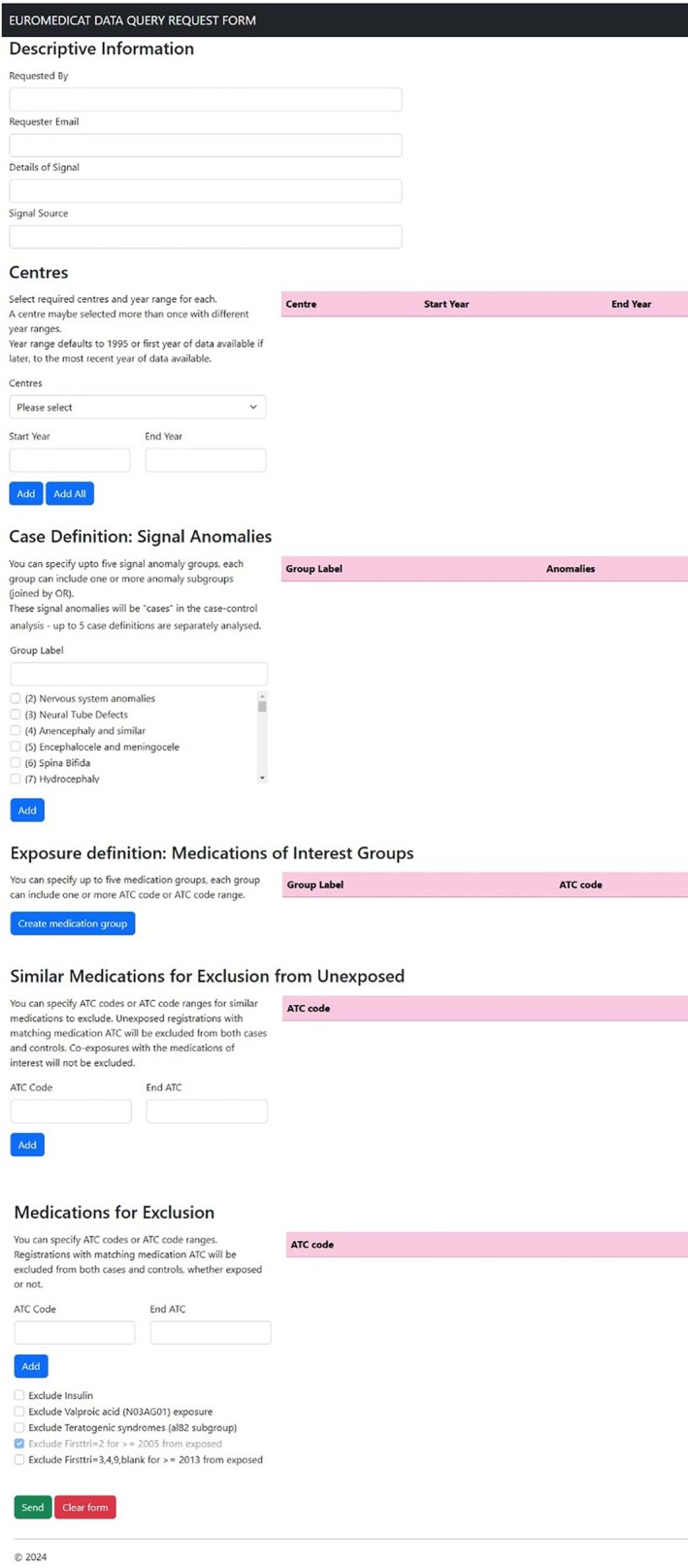
Screenshot of automated query system. The data requester enters the details of the previous signal to be tested, which are read into a standard case‐malformed control study program analyzing the central database.

An automated query system has been developed, by which any signal can be tested in the central database according to a standard protocol in a case‐malformed control design (Figure 3).

### 3.2 Cohort Studies

Cohort studies are possible for registries contributing to the distributed database which can link CA registries to data on all births in the source population as well as to data on prescriptions (eight centers, Table 1). Examples are studies of antiasthmatics and SSRIs [15, 16]. These have the advantage of giving an estimate of relative risk of all major CA combined, and can adjust for a wider set of confounder variables. Cohort studies allow study of CA to be integrated with study of other pregnancy outcomes such as small for gestational age or neurodevelopmental outcomes, as well as breastfeeding [39]. They are, however, lengthier and costlier.

### 3.3 Disease‐Specific Cohorts

Different treatments can be compared within a disease‐specific cohort. Where healthcare databases collect insufficient information about the maternal disease or treatment, a hybrid (primary‐secondary data) design can be employed. In a study of insulin analogues, EUROmediCAT registries constructed a cohort of mothers with diabetes in hospitals within registry areas by primary data collection, recording the type of insulin taken and level of glycaemic control from medical records, and then linked that cohort to the CA registry to determine outcome [40]. Although primary data collection for hybrid studies needs resources, it can offer strong scientific value relative to the investment.

### 3.4 Signal Detection Studies

The EUROmediCAT central database as a whole can be used for signal detection studies based on disproportionality, comparing the proportion of each specific CA exposed to each medication (at different levels of ATC coding [10]) to the proportion of all other CAs exposed to each medication. The main issue with such a large database is to guard against multiple testing, as many spurious statistically significant associations are expected. Statistical methodology has been refined to mitigate multiple testing, using the False Discovery Rate [41], a Double False Discovery Rate [42], Bayesian hierarchical models [43] and Bayesian Biclustering hierarchical Models [44]. These methods have similar acceptable performances and therefore it may be advantageous for clarity and ease of application to adopt the double false discovery method, which is simple to apply and performs better than just the false discovery method. The most important step after the statistical analysis is a process of expert prioritization of the signals produced [45, 46], involving expert opinion, review of the literature, biological plausibility, strength of the signals, and further analysis of the data.

The potential for conducting signal‐detection studies in the distributed database is to be explored, but is likely to be complex and initially lengthy until replicable processes are established and tested.

### 3.5 CA Case Series for New Medications

Where a medication is new to the market, there are no previous signals to test, and, initially, there are not enough exposed pregnancies for a full study, an annual case series of exposed cases may allow an evaluation in the early years of whether any specific CA (or combination of CAs) stands out in unusual proportions. The potential for these population‐based case series to be evaluated alongside spontaneous adverse event reports has not yet been realized.

### 3.6 Prevalence and Ecological Studies Evaluating Population‐Level Exposures

Prevalence studies can be used to evaluate preventive interventions e.g., the EUROmediCAT central database has been used to monitor the prevalence of valproic acid syndrome over time [47]. Ecological studies of CA prevalence in relation to pandemics (the H1N1 flu pandemic [48], and more recently the Covid‐19 pandemic) can consider the overall impact on CA prevalence (all and specific CA) of population exposure to infection, treatment, and vaccine.

### 3.7 Medication Utilisation Studies

Medication utilization studies can only be conducted using the EUROmediCAT distributed database, as data on all pregnancies/births is required. Such studies do not require the CA registry data and thus can include other databases also [5, 6, 7, 49]. These studies can be used to evaluate regulatory actions, for example, changes in prescription of valproic acid in pregnancy before and after the change in advice from EMA in 2014 [5, 49]. We also recommend that medication utilization studies be twinned with risk studies [5, 6, 7] to assess expected levels of exposure, differences between countries, changes in prescribing over time, prescribing patterns prior to and during pregnancy, and potential confounders such as maternal age. New approaches to characterizing medication utilization in pregnancy could in future lead to CA risk studies with a more sophisticated exposure classification [50].

## 4 Strengths and Limitations

The impact of the research arising from EUROmediCAT demonstrates the value of this long‐standing collaboration, combining high quality standardized CA data with a large population size and multidisciplinary expertise. For example, research on valproic acid [25], lamotrigine [34], and metformin [32] using the central database, and SSRIs [16] using the distributed database, has contributed to prescribing guidelines for pregnancy [51, 52, 53, 54]. As well as addressing the evidence gap concerning existing medicines, EUROmediCAT can continue to meet new challenges, such as pandemics [48, 55, 56], new medicines, and new vaccines.

CA registries are an essential data source contributing to both the EUROmediCAT central database and distributed database, since the quality of data on CA in healthcare databases is poor for many types of CA. Studies based on healthcare databases alone suffer from a number of limitations which include small population sizes; lack of data on TOPFA or restriction to livebirths; lack of data on CA diagnosed after the neonatal period; incomplete diagnostic information so that specific CA subgroups cannot be reliably studied and genetic syndromes cannot be excluded; and poorly validated coding of CA in healthcare databases, with low sensitivity and low positive predictive value for many conditions [57, 58, 59], poor distinction between suspected and confirmed CA diagnoses, and incomplete and biased ascertainment from surgical procedures.

Medication exposure ascertainment incompleteness or inaccuracy (particularly with regard to timing) may lead to bias, depending on study design, and/or loss of statistical power. Case‐malformed control study design mitigates against bias as similar levels of underascertainment are expected in both cases and malformed controls. Exposed cases and controls are individually checked by registries for each study using the central database to verify exposure timing in the first trimester. To the extent that inaccuracies remain, this non‐differential bias would lead to dilution of odds ratios. Cohort studies using the distributed database, containing linked prescription data, may also have some bias linked to the recording of medications which were not taken, or taken at a different time, or prescriptions issued outside the time window for analysis but taken within the time window of exposure. Given the prospective nature of the exposure data, the bias is expected to be non‐differential leading to dilution of odds ratios. While the EUROmediCAT network covers a large population, both with the central database and the distributed database, there is potential for expansion, both in the size of the European population covered (by both EUROCAT registries and healthcare databases), and its geographical and socioeconomic diversity. Several new CA registries are expected to join in 2026. There is also potential for improvement in the sources of exposure data available, for example, improved recording of medication exposure in antenatal records, and the inclusion of in‐patient hospital prescriptions in healthcare databases.

Linkage between healthcare databases with the use of civil identification numbers or healthcare identification numbers is now possible in many countries, although accurate linkage of mothers and babies is lagging behind. Some countries have provided safe ways of accessing anonymized data, but processes to obtain permission and access may be lengthy and need to be streamlined for pharmacovigilance using the distributed database. The establishment of European data safe havens where individual patient data (IPD) from all European countries can be accessed needs to occur to enable the optimum analysis of IPD across Europe.

## 5 Conclusions

EUROmediCAT presents a unique data and expert resource for tackling the enormous evidence gap regarding the safety of medication during pregnancy, contributing to the prevention of congenital anomalies, and to the opportunity for optimal treatment of pregnant women balancing the benefits and harm of medication.

## Funding

We thank the staff, past and present, of EUROCAT congenital anomaly registries, and the funders of each registry (https://eu‐rd‐platform.jrc.ec.europa.eu/eurocat/eurocat‐members/registries_en). We thank Prof Lolkje de Jong van den Berg for her inspirational collaboration establishing EUROmediCAT. We thank Karin Kallen for collaboration in EUROmediCAT regarding data from Sweden. The EUROmediCAT databases were established under EUFP7 grant agreement HEALTH‐F5‐2011‐260598. The compilation of this article was part funded by the ConcePTION project under grant agreement no. 821520 with the Innovative Medicines Initiative 2 Joint Undertaking, which receives support from the European Union's Horizon2020 research and innovation program and EFPIA.

## Conflicts of Interest

The authors declare no conflicts of interest.

## Appendix 1 EUROmediCAT Central Database: Data Dictionary

The following variables are the EUROmediCAT central database variables as described in the EUROCAT Data Dictionary of EUROCAT Guide 1.5 updated version November 2025 Guidelines for data registration|European platform on rare disease registration. Previous versions of the EUROmediCAT data dictionary (e.g., corresponding to EUROCAT Guide 1.4) may be specified in EUROmediCAT publications, and can be found on the EUROCAT website. There are very few changes between EUROCAT Guide 1.4 and Guide 1.5 in the variables used in the EUROmediCAT dataset. Note that the Variable Number is the EUROCAT Variable number, but not all EUROCAT variables are in the EUROmediCAT dataset; hence some missing variable numbers in the sequence.

**Table:**

Baby and Mother (core variables shaded blue)			
Variable number	Variable name	Explanation and instructions	Code
1	CENTRE	centre number	Code allocated by central registry
2	NUMLOC	local id Each case has a unique identification. This number is a maximum of 11 characters long, consisting of numbers, letters or both. ID numbers should not repeat themselves in different years.	Up to 11 digits
3	BIRTH_DATE	date of birth Please enter dates in one of the following format: dd/mm/yyyy, yyyy/mm/dd, yyyy/mm/xx, yyyy/xx/xx. Old formats are also accepted: ddmmyy, 99mmyy and 9999yy. Please make sure to choose one format and stick to it.	Day, month, year. xx = Not known for day and month. DO NOT TRANSMIT RECORDS IF YEAR OF BIRTH IS NOT KNOWN.
4	SEX	sex Indicate chromosomal sex, if known, in case of ambiguous genitalia code malformations in variables 32–47. Indicate indeterminate sex in case of ambiguous genitalia with unknown or abnormal sex chromosome complement. If sex could not be determined at autopsy due to maceration, very small fetus, or other problems, indicate as “not known”.	1 = Male. 2 = Female. 3 = Indeterminate. 9 = Not known.
5	NBRBABY	number of babies/fetuses delivered Fill out a separate form for each malformed baby/fetus in a multiple set. Only one form is to be completed for conjoined twins (Siamese). The code is “2” for a conjoined twin unless another baby was delivered at the same time (code “3”). Conjoined twins have a specific ICD/BPA code, to be coded under “MALFO1” (variable 32). Give full description of the type of conjoined twinning in MALFO1 text field (variable 33). Any other anomalies are coded in variables 32–47. Notes. If code 8 is used, please specify in variable sp_twin the gestational age at which last known to be a multiple pregnancy and/or first known to be a singleton. The purpose of this coding system is to allow us to distinguish malformed cases which would have civil registration as singleton births from malformed cases which would have civil registration as multiple births. Please specify the sex and outcome (live, still) of the malformed/non‐malformed co‐twin and zygosity.	1 = Singleton. 2 = Twins. 3 = Triplets. 4 = Quadruplets. 5 = Quintuplets. 6 = Sextuplets or more. 7 = Multiple birth, number of babies not known. 8 = Singleton at time of delivery/termination, but known to have been a multiple pregnancy at an earlier stage in pregnancy. 9 = Not known.
6	SP_TWIN	specify twin type of birth (malformed and non‐malformed), like or unlike sex, zygosity.	Free text.
7	NBRMALF	number of malformed in multiple set. To be completed for multiple delivery only. Remember to give local ID of co‐twin in SIB1 field (variable 92) if more than one malformed.	1 = One. 2 = Two. 3 = Three. 4 = Four. 5 = Five. 6 = Six or more. 9 = Not known.
8	TYPE	type of birth. Birth with the type of birth not known should be transmitted to EUROCAT, but will be excluded from routine EUROCAT analysis. EUROCAT includes all live births, fetal deaths with gestational age (GA) ≥ 20 weeks, and terminations of pregnancy (at any gestational age) after prenatal diagnosis of malformation (TOPFA). Fetal deaths with gestational age (GA) < 20 weeks (code = 3) may be reported to EUROCAT but will not be included in prevalence data. All cases MUST have been confirmed as having a MAJOR congenital anomaly (see exclusion list, chapter 3.2). The distinction between stillbirth and spontaneous abortion should follow the definitions in use in your country (to be specified in your Registry Description). There is usually a lower gestational age limit or birthweight limit for stillbirths. This varies from country to country. Below this limit, fetal deaths are called spontaneous abortions. Terminations of pregnancy refer to cases where a prenatal diagnosis was made of malformation in a live fetus and the pregnancy was then terminated. If the fetus died spontaneously in utero either before or after prenatal diagnosis of malformation then it should be coded as spontaneous abortion or stillbirth, not as termination of pregnancy. If termination was performed for other reasons than malformation, the case should not be transmitted to Central Registry. This means that early terminations where there was no suspicion of malformation before termination should be excluded from the case files. Stillbirths or perinatal deaths resulting from termination of pregnancy following prenatal diagnosis must be coded as terminations (value = 4), irrespective of civil registration status. For a non‐natural fetal reduction in a multiple pregnancy where one fetus is malformed, code 4 (in that case gestlength = gestational age at reduction; date of birth = date of reduction; and code carefully all multiple birth variables).	1 = Live birth. 2 = Stillbirth. 3 = Spontaneous abortion. 4 = TOPFA. 9 = Not known.
10	WEIGHT	birth weight. Give weight in grams.	9999 = Not known. (Do not use 99 or 999 for “Not Known” as this will be considered the birth weight).
11	GESTLENGTH	length of gestation in completed weeks. Give best estimate based on last menstrual period (LMP) and/or ultrasound determination. If the case is the result of fetal reduction give GA at feticide. Check GA below 10 weeks or above 43 weeks.	99 = Not known.
12	SURVIVAL	survival beyond 1 week of age Yes = Child known to be alive after 1 week. No = Child known to have died before or during the first week (including stillbirths and abortions). Alive at discharge < 1 week refers to cases that are alive at discharge from maternity units before 1 week of age. Please specify in your Registry Description the day when discharge from maternity units usually takes place. If survival at 1 week is unknown, but survival at discharge from maternity unit less than 1 week is known, use the latter. The definition of the first week of life varies between countries. Follow your country's perinatal mortality definition and specify this in your Registry Description. Not known = Not known if a child has died during first week.	1 = Yes. 2 = No. 3 = Alive at discharge < 1 week. 9 = Not known.
15	AGEMO	age of the mother at delivery. In completed years at the time of delivery. If only the year of birth is available, assume that the mother was born on 30 June.	99 = Not known.
16	BMI	maternal body mass index. Enter BMI (rounded to one decimal place). The DMS will also allow entry of maternal height (in centimeters) and weight (in kilograms) and calculate BMI automatically. Values measured at first antenatal visit are preferred, but pre‐pregnancy self‐reported values may be given. If the mother is known to be obese, enter code for obesity E660 in maternal illness before pregnancy (variables 61–63). BMI was a new variable in Guide 1.4. If any registry has information on maternal BMI for previous years, EUROCAT is interested in collecting this information from 2005 onwards.	Rounded to one decimal place. Expected range 15–50. 96 = exact BMI not known but BMI < 18.5. 97 = exact BMI not known but 18.5 to < 30. 98 = exact BMI not known but > = 30. 99 = Not known.
18	TOTPREG	total number of previous pregnancies. NOTE—The current reported pregnancy is NOT included. Include all previous abortions whether spontaneous or induced. Multiple pregnancies count as 1 in the total.	00 = None. 01 = One. 02 = Two. 03 = Three, et cetera. 20 = Twenty or more. 99 = Not known.

Diagnosis (core variables shaded blue)			
Variable Number	Variable Name	Explanation and Instructions	Code
19	WHENDISC	when discovered. When the baby was first suspected of having a congenital anomaly. For prenatal diagnosis: when a major congenital anomaly was first suspected (EXCLUDING soft markers except if nuchal translucency indicates a very high risk followed by confirmation of diagnosis at delivery/termination). If a prenatal diagnosis is made when a fetus is dead code 1 (for stillbirths) or 7 (for spontaneous abortions). For live births: when the first suspicion of an anomaly was at death OR at *post mort*em, when discovered is the age at death (e.g., at birth, < 1 week, 1–4 weeks etc.). For stillbirths: when the first suspicion of an anomaly was at birth OR at *post mortem*, when discovered is at birth (e.g., Code = 1). All cases MUST have been confirmed as having a MAJOR congenital anomaly (see exclusion list, chapter 3.2). EUROCAT accepts a positive NIPT for trisomy 13, 18 and 21 without a full karyotype. Please also complete variables 12 “SURVIVAL”, 13 “DEATH‐DATE”, 20 “CONDISC” and 28 “PM”.	1 = At birth. 2 = Less than 1 week. 3 = 1–4 weeks. 4 = 1–12 months. 5 = Over 12 months. 6 = Prenatal diagnosis in live fetus. 7 = At abortion (spontaneous). 9 = Not known. 10 = Postnatal diagnosis, age not known.
21	AGEDISC	if prenatally diagnosed, gestational age at discovery in completed weeks. GA as defined in variable GESTLENGTH. Gestational age at which the fetus was first suspected to be malformed (EXCLUDING soft markers). Indicate time of examination rather than time when the result was known. If no prenatal diagnosis please leave blank.	99 = Not known.
24	KARYO	karyotype of infant/fetus. Specify the result in variable 25. Array results count as a karyotype test. Report only clearly pathogenic variants and if uncertain, include only copy number variants (CNVs) (duplications or deletions) larger than 1 MB. Only report cases with de novo CNVs unless the parent in familial cases also has clinical manifestations of the condition (dysmorphic features or congenital anomalies). (Coding Committee 2015). If performed and results known, please specify (according to the latest ISCN edition). “Probe test performed” refers to FISH, PCR, NIPT or other analyses restricted to specific chromosomal regions. “Failed” refers to a technical failure where a repeat examination could not be done and the karyotype is therefore unknown.	1 = Performed, result known. 2 = Performed, results not known. 3 = Not performed. 4 = Probe test performed. 8 = Failed. 9 = Not known.
25	SP_KARYO	specify karyotype or chromosomal microarray. 47, XY, +21. 46, XX, del (2) (p13p23)*mat Description: An interstitial deletion resulting from mal‐segregation of a maternal insertion* 46, XY, der (5)ins (5;2) (q31;p23p13) mat *Description*: *A derivative chromosome 5 resulting from mal‐segregation of a maternal insertion from chromosome 2*. arr (1–22) × 3, (X) × 2, (Y) × 1 *Description: Microarray analysis shows triploidy 69, XXY* arr (8) × 3, (21) × 3 *Description: Microarray analysis shows a single copy gain of chromosomes 8 and 21*. 46, XY.rsa (13, 18, 21) × 2, (X, Y) × *1 Description: normal chromosomes, only chromosome 13, 18, 21, X and Y investigated* rsa 22q11.2 (“kit name”) × 1 *Description: microdeletion 22q.11.2 diagnosed by multiple ligation probe amplification method (MLPA)*.	Free text.
26	GENTEST	GENETIC TEST. For syndromes and single gene disorders, a genetic test may have confirmed the clinical diagnosis either prenatally or postnatally. Please complete for these cases. Karyotype should still be completed as per variables 24 & 25. If any registry has this information for previous cases, EUROCAT is interested in collecting this information from 2005 onwards. If the test is performed but the result not yet known, please wait for the result before reporting.	1 = specific genetic test positive. 2 = specific genetic test negative. 3 = Specific genetic test not Performed. 9 = Not Known if genetic test is performed or result not known.
27	SP_GENTEST	specify type of genetic test. Give method used and the result of the test (type of mutation and which gene). Examples: Single gene analysis, exome sequencing, gene panel analysis, whole genome sequencing.	Free text.
28	PM	*post mortem* examination. If performed, record the malformation(s) discovered in the “malformation” section in the form. If other findings, record in the “general remarks” space (variable 103). “Results known” means that the autopsy record has been reviewed by the registry. “Results not known” means that the autopsy record was not available to the registry. “Macerated fetus” means that although a *post mortem* was performed, maceration of the fetus prevented a full protocol from being followed.	1 = Performed, results known. 2 = Performed, results not known. 3 = Not performed. 4 = Macerated fetus. 9 = Not known.
30	SYNDROME	syndrome or association. All cases MUST have been confirmed as having a MAJOR congenital anomaly (see exclusion list, chapter 3.2). Use this variable for genetic syndromes, skeletal dysplasias, hereditary skin disorders teratogenic syndromes, associations, microdeletions and chromosomal anomalies. Refer to EUROCAT Guide on syndromes. Give the name of syndrome or association in text variable 31. All the anomalies observed by the local clinician should be coded in the remaining boxes for malformations. If not a recognized syndrome or association, leave blank. When 2 syndromes are present in the same subject, code the more important one in the syndrome variables 30 and 31, and include the other one in variables 32 and 33 MALF01. Ensure karyotype information is given in variables 24 and 25 and that information on genetic tests are given in variable 26 and 27. Mention in variable 28 if the autopsy report has been reviewed, where appropriate. Local registries are advised to keep photographs and x‐ray images of all syndrome cases if possible, as the diagnosis might be established on the basis of specific facial dysmorphism.	ICD 10. First 4 digits are ICD10. 5th digit = BPA supplement or leave blank.
31	SP_SYNDROME	specify syndrome. Written text description of the ICD10 code in variable 30.	
32	MALFO1	malformation. All cases MUST have been confirmed as having a MAJOR congenital anomaly (see exclusion list, chapter 3.2). A baby/fetus with ONLY minor anomalies (see exclusion list, chapter 3.2) should not be transmitted to Central Registry. This rule does not concern established syndromes (e.g., Down, Beckwith Wiedemann or Prader Willi syndrome without a major congenital anomaly are to be submitted). When a major anomaly is present, code both major and minor anomalies. In the case of conjoined twins, give a full description in text in variable 33. Up to 8 malformations can be coded—if more than 8 are present, specify additional anomalies in the text variable for the 8th anomaly (text variable 47 SP_MALFO8). Include in the 8 specified codes the most important ones, or those tabulated in EUROCAT Reports. Give written description of the malformations available in malformation text variables 33, 35, 37, 39, 41, 43, 45 and 47.	ICD 10. First 4 digits are ICD. 5th digit = BPA classification OR leave blank.
33	SP_MALFO1	specify malformation	Free text
34	MALFO2	as malfo1	As MALFO1
35	SP_MALFO2	specify malformation	Free text
36	MALFO3	as malfo1	As MALFO1
37	SP_MALFO3	specify malformation	Free text
38	MALFO4	as malfo1	As MALFO1
39	SP_MALFO4	specify malformation	Free text
40	MALFO5	as malfo1	As MALFO1
41	SP_MALFO5	specify malformation	Free text
42	MALFO6	as malfo1	As MALFO1
43	SP_MALFO6	specify malformation	Free text
44	MALFO7	as malfo1	As MALFO1
45	SP_MALFO7	specify malformation	Free text
46	MALFO8	as malfo1	As MALFO1
47	SP_MALFO8	specify malformation	Free text
57	OMIM	omim/type of mendelian inheritance To be coded by medical geneticist or after advice from medical geneticist. For reporting OMIM refer to EUROCAT Syndrome Guide. The first digits may be filled in without the rest of the code if the full OMIM code is not known. Full codes can be found on the OMIM website. http://www.ncbi.nlm.nih.gov/omim/	
58	ORPHA	This code is to be used for rare diseases including congenital anomalies, chromosomal, teratogenic and genetic syndromes. Use the link and enter the name of the condition. If more than one code/disease appears, select the most specific ORPHAcode. If you do not have specific information about genetic background or phenotype, select the most general ORPHAcode. https://www.orpha.net/consor/cgi‐bin/Disease.php?lng=EN	

Exposure (core variables shaded blue)			
Variable Number	Variable Name	Explanation and Instructions	Code
61	ILLBEF1	illness before pregnancy 1. Record any maternal illness whether chronic or acute with onset before pregnancy that may affect fetal development (e. g. childhood cancer, metabolic and endocrine disease, severe congenital anomaly). Code according to ICD10. The codes mentioned below are only examples (non‐exhaustive list). illbef1 and illbef2 does NOT imply the disease in illbef1 is more important/severe than the disease in illbef2 or illbef_extra. If a mother has pregestational diabetes this should always be entered in variable 64 (MATDIAB). Historic maternal diseases may be coded using the Z‐chapter in ICD10, example Z853 “Personal history of breast cancer”. Any additional details may be entered in the general comments section (variable 103). Do not insert the decimal point in the code (e. g. Code E05.0 as E050). Examples, non‐exhaustive list: Hyperthyroidism E050–E059. Hypothyroidism E000–E039. Diabetes Type 1 E100–E109. Diabetes Type 2 E110–E119. Obesity E660–E669. If maternal BMI ≥ 30 give code for obesity. Metabolic disorders E700–E889. Anorexia/eating disorders F500–F509. Depression F320–F339. Epilepsy G400–G409. Hypertension I100–I159. Asthma J450–J459. Chronic alcoholism F102. Drug addict F112–F122–F132–F142. COVID‐19 (only report if within 3 months before pregnancy): B342 (previously U071 and U072 were used).	ICD 10. 0 = No illness. 1 = Yes, but no information available. 9 = Not known.
62	ILLBEF2	illness before pregnancy 2—as for illbef1.	
63	ILLBEF_EXTRA	other illness(es) before pregnancy. This field is only to be used if “illnesses before pregnancy” fields 1 and 2 have already been filled, to record additional maternal illnesses before pregnancy. Follow the coding instructions of illbef1. Please enter the ICD code in the following format: <ICD code>. If more than one additional illness before pregnancy is to be reported for a single case, then enter the ICD codes in the illbef_extra field as follows: <ICD code><ICD code>. For example, a case with hypothyroidism and depression exposure is entered in the illbef_extra field as: <E000> <F320>.	
64	MATDIAB	MATERNAL PREGESTATIONAL DIABETES. This variable is specifically for pregestational diabetes. Gestational diabetes is dealt with under the ‘illness during pregnancy’ variable (variable 65). Type 1 diabetes: characterized by hyperglycaemia due to an absolute deficiency of the insulin hormone produced by the pancreas. An HbA1c of 48 mmol/mol is recommended as the cut‐off point for diagnosing diabetes. Type 2 diabetes: characterized by hyperglycaemia due to a defect in insulin secretion. An HbA1c of 48 mmol/mol is recommended as the cut‐off point for diagnosing diabetes. *Maturity Onset Diabetes in the Young (MODY) displays an autosomal dominant pattern of inheritance. An HbA1c of 48 mmol/mol is recommended as the cut‐off point for diagnosing diabetes. Impaired Glucose Intolerance is a state of higher than normal blood (or plasma) glucose concentration, but less than the diagnostic cut‐off for diabetes. Diagnosed before pregnancy. Diagnosed by fasting plasma glucose from 6.1–6.9 mmol/L (WHO criteria).	1 = Yes, type 1 diabetes (IDDM). 2 = Yes, type 2 diabetes (NIDDM). 3 = Yes, type MODY* (all types). 4 = Yes, type not known. 5 = No, but impaired glucose intolerance. 6 = No pregestational diabetes. 9 = Not known.
65	ILLDUR1	illness during pregnancy. Record maternal illnesses with chronic or acute onset during the first 20 weeks of pregnancy, including asymptomatic maternal infections. For gestational diabetes include at any point in pregnancy. illdur1 and illdur2 does NOT imply the disease in illdur1 is more important/severe than the disease in illdur2 or illdur_extra. This variable aims to capture the maternal illnesses that may affect fetal development. For early pregnancy complications use the O chapter in ICD10. For maternal infections, use chapters A and B (4 digits). Fetal infections and associated malformations should be coded under syndrome and malformation 1–8 codes (variable 30–47). Examples (non‐exhaustive list): Gestational diabetes O244—O249. Hemorrhage early pregnancy O200—O209. Hyperemesis O210—O219. Fever R502—R509. Hypertension I100—I159. Depression F320—F339. CMV B250—B259. HIV (AIDS) B200—B249. Influenza J100—J119. Mumps B260—B269. Rubella B060—B069. Syphilis A530—A539. Toxoplasmosis B580—B589. Varicella (Chickenpox) B010—B019. Viral hepatitis B190—B199. Zika virus A925. Drug poisoning T360‐T509. COVID‐19 B342 (previously U071 and. U072 were used).	ICD 10. 0 = No. 1 = Yes, but no information available. 9 = Not known.
66	ILLDUR2	illness during pregnancy. as for illdur1.	
67	ILLDUR_EXTRA	extra illness during pregnancy. This field is only to be used if “illnesses during pregnancy” fields 1 and 2 have already been filled, to record additional maternal illnesses during pregnancy. Follow the coding instructions of illdur1. Please enter the ICD code in the following format: <ICD code>. If more than one additional illness during pregnancy is to be reported for a single case, then enter the ICD codes in the illdur_extra field as follows: <ICD code><ICD code>. For example, a case with CMV and depression exposure is entered in the illdur_extra field as: <B250><F320>.	
68	FOLIC_G14	folic acid supplementation. Recommend to your local maternity hospitals or midwives to collect these data. Folic acid supplementations include folic acid only tablets, a multivitamin preparation which contains folic acid or contraceptive pills which contain folic acid. If the folic acid dose is high (≥ 4 mg), please add the code B03BB01 in the drugs variable.	1 = Folic acid taken pre‐ and post‐conceptionally. 2 = Folic acid taken only post‐conceptionally. 3 = Folic acid not taken. 4 = Folic acid taken, timing unknown. 9 = Not known if folic acid taken.
69	FIRSTTRI	FIRST TRIMESTER MEDICATION. “Yes” means that the data sources clearly state that medication was taken in the first trimester. “No” means that the data sources clearly state that no medication was taken in the first trimester. Medications taken in the 2nd or 3rd trimester only should not be added to the DMS. “Medication taken but timing unknown” means that the usual data sources stated that medication was taken but the timing of use was not stated. “Not Known” means that the usual data sources were not found. Only fill in DRUGS1‐5 and EXTRADRUGS if you have coded FIRSTTRI = 1 (Yes medication taken) or = 4 (Medication taken, but timing unknown). If you have coded FIRSTTRI = 2 (no medication taken), FIRSTTRI = 9 (unknown), there shouldn't be any ATC codes in any of the DRUGS variables. Include any medication that was taken by the mother during the first trimester of pregnancy (from the 1st day of the last menstrual period up to the 12th week of gestation). Medication with long elimination half time and taken before conception should be included (e.g., Acitretin, Etretinate, etc.). Use of folic acid (either as folic acid only tablets or a multivitamin preparation which contains folic acid) should be registered in the folic acid variable, but if the folic acid dose is high (≥ 4 mg), please register FIRSTRI = 1. Do not include usual vitamins and mineral supplementation, but include unusual intakes of vitamins or minerals (e.g., Vitamin A mega doses). Only medication taken at physiologic doses should be included. Whilst FIRSTTRI is a new variable introduced in Guide 1.4 (for cases born from 2013 onwards). If any registry has this information for previous cases, EUROCAT is interested in collecting this information from 2005 onwards. *Note: A code “3 = undetermined” was in use until birth year 2022. It has been discontinued but there are cases with the code = 3 in the database for some registries. It is advised to recode the variable as “9 = Not know” when analysing the data*.	1 = Yes, medication taken in first trimester. 2 = No medication taken in first trimester. 4 = Medication taken, but timing unknown. 9 = Not Known.
70	DRUGS1	drugs—7 digits maximum. Record any drug taken by the mother during the first trimester of pregnancy (from the 1st day of the last menstrual period up to the 12th week of gestation). Drugs with long elimination half time and taken before conception should also be recorded (e.g., Acitretin, Etretinate, etc.). If it is not known in which trimester the drug was taken, and this information cannot be obtained, code it but write in the space for comments that it is not sure whether the drug was taken in the first trimester. Use ATC‐coding and use as many digits as possible (from 3 to 7). Website http://www.whocc.no/atcddd/. Do not record usual vitamins and mineral supplementation, but record unusual intakes of vitamins or minerals (e.g., Vitamin A mega doses, folic acid dose ≥ 4 mg). The ATC coding system does not have a code for alternative drugs or herbs. If these are used, give the main code Z. ATC example: N03A: antiepileptic drug. N03AF01: carbamazepine. Details on the dosage and timing should be given in text variable 71. Do not forget to mention in the appropriate section (disease during or before pregnancy) the indication for drug use. Only drugs taken at physiologic doses to be recorded. If a drug overdose or self‐poisoning, this MUST be explained in the drug description.	
71	SP_DRUGS1	specify drug exposures	Free text.
72	DRUGS2	as for drugs1. Please give details in text variable 73 SP_DRUGS2.	As for DRUGS1.
73	SP_DRUGS2	specify drug exposures	Free text.
74	DRUGS3	as for drugs1 Please give details in text variable 75 SP_DRUGS3.	As for DRUGS1
75	SP_DRUGS3	specify drug exposures	Free text.
76	DRUGS4	as for drugs1 Please give details in text variable 77 SP_DRUGS4.	As for DRUGS1
77	SP_DRUGS4	specify drug exposures	Free text.
78	DRUGS5	as for drugs1 Please give details in text variable 79 SP_DRUGS5.	As for DRUGS1.
79	SP_DRUGS5	specify drug exposures	Free text.
80	EXTRA_DRUGS	extra drugs This field is only to be used if drug fields 1–5 have already been filled. Record any drug taken by the mother during the first trimester of pregnancy (from the 1st day of the last menstrual period up to the 12th week of gestation). Drugs with long elimination half time and taken before conception should also be recorded (e.g., Acitretin, Etretinate, etc.). If it is not known in which trimester the drug was taken, and this information cannot be obtained, code it but write in the space for comments that it is not sure whether the drug was taken in the first trimester. Use ATC‐coding and use as many digits as possible (from 3 to 7). Website http://www.whocc.no/atcddd/. Do not record usual vitamins and mineral supplementation, but record unusual intakes of vitamins or minerals (e.g., Vitamin A mega doses). The ATC coding system does not have a code for alternative drugs or herbs. If these are used, give the main code Z. ATC example: N03A: antiepileptic drug. N03AF01: carbamazepine. Details on the dosage and timing should be given in the drug description. Do not forget to mention in the appropriate section (disease during or before pregnancy) the indication for drug use. Only drugs taken at physiologic doses to be recorded. If a drug overdose or self‐poisoning, this MUST be explained in the drug description. Please enter the ATC code and text description in the following format: <ATC code|text description>. If more than one extra drug is to be imported for a single case, then enter the ATC codes in the extra drugs field as follows: <ATC code|text description> < ATC code|text description>. For example a case with valproate and lamotrigine exposure is entered in the extra_drugs field as: <N03AG01|Valproate> < N03AX09|Lamotrigine>.	

Family History (core variables shaded blue).			
Variable Number	Variable Name	Explanation and Instructions	Code
89	SIBANOM	sibs with anomalies. If the sibling (including twin) was notified to EUROCAT fill in variables 91–94 below. Make sure that the local identification numbers given correspond to those in the central database; otherwise give more information in text here. If previous siblings were not notified to EUROCAT specify in text SP_SIBANOM the year of birth and malformations of each sibling. If one sibling has both the same anomaly and a different anomaly, code under “same”. If one sibling has the same anomaly and another sibling has a different anomaly, code under “same and other”. Always give details in text variable 90 SP_SIBANOM.	1 = Same. 2 = Other. 3 = Same and other. 4 = No. 9 = Not known.
90	SP_SIBANOM	specify type of anomaly of siblings.	Free text.
91	PREVSIB	previous malformed siblings notified to eurocat. If yes, give the local ID number in variables SIB1, SIB2 or SIB3 (variables 92–94). Include malformed co‐twins or siblings from the same pregnancy, irrespective of birth order within multiple set. Exclude, conjoined twin.	1 = Yes. 2 = No. 9 = Not known.
92	SIB1	sib local id number notified to the central registry. Enter here also the code numbers of co‐twins or siblings from the same pregnancy, irrespective of birth order within multiple sets. Leave blank if no previous siblings notified to EUROCAT.	Local ID.
93	SIB2	as sib1.	Local ID.
94	SIB3	as sib1.	Local ID.
95	MOANOM	mother's family with anomalies. Include mother herself as well as mother's family. Specify type of anomaly in written text and relation to the infant. If the etiology is known, “same” means the same etiology, even if the spectrum of malformations present is slightly different. If the etiology is unknown or multifactorial, “same” is a matter of judgment by a qualified coder, but full specification of the anomaly should be given, whether other or the same. “Same and other” refers to two different relatives. If a relative has both the same and another anomaly, code “same”. Restrict the family to first, second and third degree relatives (mother, father, siblings, grandparents, aunts, uncles, half‐siblings, first cousins). Always give details in text variable 96 SP_MOANOM.	1 = Same. 2 = Other. 3 = Same and other. 4 = No. 9 = Not known.
96	SP_MOANOM	specify type of anomaly and describe the malformation	Free text.
97	FAANOM	father's family with anomalies As MOANOM. Please give details in text variable 98 SP_FAANOM.	As MOANOM.
98	SP_FAANOM	specify type of anomaly and describe the malformation	Free text.

Sociodemographic (core variables shaded blue).			
Variable Number	Variable Name	Explanation and Instructions	Code
99	MATEDU	maternal education Refer to International Standard Classification of Education 2011 (http://uis.unesco.org/sites/default/files/documents/international‐standard‐classification‐of‐education‐isced‐2011‐en.pdf). Assign according to the highest level of education completed (or for full‐time students, level in progress). Elementary and lower secondary refers to the period of compulsory education, usually to age 15/16. Upper secondary refers to the last two school or college years (usually to age 18) preparing students for tertiary education or the workforce. Tertiary refers to Bachelor's degree (English), Diploma (German), License (French) or equivalent, and to higher degrees (e.g., doctorates), or to other forms of higher education.	1 = Elementary and lower secondary. 2 = Upper secondary. 3 = Tertiary. 9 = Not known.
100	SOCM	socioeconomic status of mother Current or last occupation. Upper non‐manual—professionals, administrators and managers for example, doctor, architect, lawyer, banker, manager, teacher, nurse, performer. Lower non‐manual—routine non‐manual for example, book‐keeper, salesman, receptionist, secretary, computer operator, clerk, waiter. Skilled manual—cook, butcher, carpenter. Unskilled manual—semi and unskilled manual for example, factory worker, driver, agricultural worker, porter. Self‐employed/artisan—owner of shop, restaurant or hotel, independent artisan. Farmer—for example, self‐employed farmer or fisherman. If code 8 (“other/student”), please specify in text in space for general comments (variable 103). For further information see: http://uis.unesco.org/sites/default/files/documents/international‐standard‐classification‐of‐education‐isced‐2011‐en.pdf	1 = Upper non‐manual. 2 = Lower non‐manual. 3 = Skilled manual. 4 = Unskilled manual. 5 = Self‐employed/artisan. 6 = Farmer. 8 = Other/student. 9 = Not known.
101	SOCF	socioeconomic status of father. As SOCM.	0 = Single mother, no father recorded. 1 = Upper non‐manual. 2 = Lower non‐manual. 3 = Skilled manual. 4 = Unskilled manual. 5—Self‐employed/artisan. 6 = Farmer. 8 = Other/student 9 = Not known.
102	MIGRANT	migrant status. This variable is included to allow assessment of the extent to which services such as prenatal screening are reaching migrants. It does not ask for ethnicity. If code 4, give text details in the general comments section (variable 103).	1 = Mother migrated from outside EU during pregnancy. 2 = Mother migrated from outside EU during adult life (from age 18). 3 = Mother not a migrant as defined in 1 or 2. 4 = Other (specify in text). 9 = Not known.

General Comments (core variables shaded blue)			
Variable number	Variable name	Explanation and instructions	Code
103	GENREM	general additional comments	Free text.

Last update:

13.08.2025

## Appendix 2 Data Quality Links for EUROmediCAT Central Database

EUROCAT Data Quality Indicators: DQI‐List‐of‐Data‐Quality‐Indicators‐since‐2012.pdf.

Most recent Data Quality Indicators 2019–2023: 20250904_DQI_2019‐2023.pdf.

Missing Values Frequency Tables 2019–2023: Missings_AllFull_2019‐2023.pdf.

## Appendix 3 Links to Databases Contributing to the EUROmediCAT Distributed Database, and to Data Catalogs and Lists of Evidence Elements and Information Items Required

**Table:**

Name of database	Links to databases
EUROmediCAT Central Database	https://www.euromedicat.eu/data/databases‐centraldatabase
EUROmediCAT – Distributed Database	https://www.euromedicat.eu/data/databases‐distributeddatabase
Funen, Denmark	National health registers ‐ The Danish Health Data Authority
Finland	Register of Congenital malformations ‐ THL Drugs and pregnancy ‐ THL Databases and registers ‐ Fimea
Haute Garonne, France (EFEMERIS)	www.efemeris.fr EMA RWD Catalogue : EFEMERIS https://catalogues.ema.europa.eu/search?search_api_fulltext=efemeris&conjunction=OR&f%5B0%5D=content_type%3Adarwin_data_source
Norway	Medical Birth Registry of Norway ‐ purpose and responsibilities ‐ NIPH Overview of health registries at FHI ‐ NIPH Legemiddelregisteret (LMR) ‐ FHI
Sweden	Startpage ‐ Socialstyrelsen
England (NCARDRS), UK	Congenital anomalies and rare diseases ‐ NDRS
Wales, UK	SAIL databank https://saildatabank.com/ CARIS – Congenital Anomaly and Information Service, Public Health Wales https://phw.nhs.wales/services‐and‐teams/caris/about‐caris/
Valencian Region, Spain	Rare Diseases | Fisabio Data Resource Profile: The Valencia Health System Integrated Database (VID) | International Journal of Epidemiology | Oxford Academic
Emilia Romagna, Italy	https://www.euromedicat.eu/data/databases‐distributeddatabase
Tuscany, Italy	https://www.euromedicat.eu/data/databases‐distributeddatabase
EMA Catalog of Real World Data Sources	https://catalogues.ema.europa.eu/catalogue‐rwd‐sources
EUROmediSAFE Inventory	https://www.euromedicat.eu/research/euromedisafe/euromedisafeinventory EUROmediSAFE Inventory_Finalv2_2018_07_06.pdf
ConcePTION Data Catalog	https://vac4eu.molgeniscloud.org/conception/catalogue/#/ Spreadsheet containing all additional data sources for the ConcePTION Data Source Catalogue (D1.1)

The evidence elements needed to conduct medication safety studies are described in: ConcePTION: Core evidence elements for generating medication safety evidence for pregnancy using population‐based data (D1.2). The information items in databases that are needed for medication safety studies are described in ConcePTION Deliverable 1.1. Spreadsheet containing all additional data sources for the ConcePTION Data Source Catalogue (D1.1).

## Appendix 4 Information Items

Each EUROmediCAT protocol for a study of a specific medication class using the Distributed Database would begin by assessing whether the medication is reliably recorded in the data sources available (according to healthcare setting in which prescribed, public/private, prescriber specialty, prescription only or OTC, years).

The core information items for medication safety studies relating to congenital anomaly risk are:

- Pregnancy timing (date and gestational age at delivery).
- ATC code.
- Prescription timing, dosage/quantity, prescriber specialty (especially where used for multiple indications), co‐prescribing.
- Indication for prescribing.
- Major congenital anomalies.
- Other infant variables: gestational age, birthweight, type of birth (live, still, TOPFA), sex, twin/singleton.
- Other maternal variables (age, parity, socioeconomic/education status).

Potentially relevant confounders (study‐specific): Folic acid, smoking, alcohol, BMI.
